# *trans*-Dibromidotetra­kis­(pyridine-κ*N*)ruthenium(II)

**DOI:** 10.1107/S1600536813000871

**Published:** 2013-01-16

**Authors:** Xiu-Li Wu, Ru-Fei Ye, Ai-Quan Jia, Qun Chen, Qian-Feng Zhang

**Affiliations:** a Department of Applied Chemistry, School of Petrochemical Engineering, Changzhou University, Jiangsu 213164, People’s Republic of China; b Institute of Molecular Engineering and Applied Chemistry, Anhui University of Technology, Ma’anshan, Anhui 243002, People’s Republic of China

## Abstract

The title complex, [RuBr_2_(C_5_H_5_N)_4_], contains two independent complex mol­ecules in each of which the Ru^II^ atom is located on a site of 222 symmetry and has a distorted octa­hedral coordination geometry with four pyridine N atoms and two Br atoms. The Br aroms are *trans*-disposed as a result of symmetry.

## Related literature

For background to ruthenium complexes: see: Pagliaro *et al.* (2005 ▶); van Rijt & Sadler (2009 ▶); Wu *et al.* (2009 ▶); Zhang *et al.* (2005 ▶). For related structures, see: Mirza *et al.* (2003 ▶); Wong & Lau (1994 ▶); Zhang *et al.* (2006 ▶). For a description of the Cambridge Structural Database, see: Allen (2002 ▶).
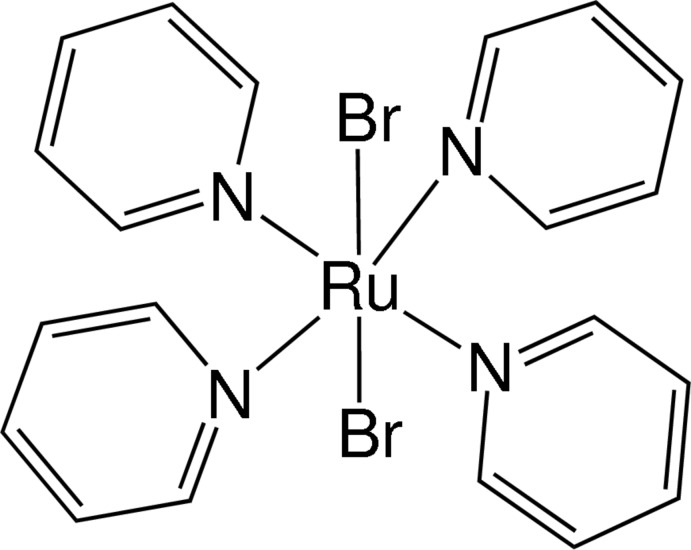


## Experimental

### 

#### Crystal data

[RuBr_2_(C_5_H_5_N)_4_]
*M* *_r_* = 577.29
Orthorhombic, 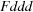

*a* = 16.830 (4) Å
*b* = 22.032 (5) Å
*c* = 23.221 (5) Å
*V* = 8610 (3) Å^3^
*Z* = 16
Mo *K*α radiation
μ = 4.45 mm^−1^
*T* = 296 K
0.22 × 0.18 × 0.13 mm

#### Data collection

Bruker APEXII CCD diffractometer
Absorption correction: multi-scan (*SADABS*; Sheldrick, 1996 ▶) *T*_min_ = 0.441, *T*_max_ = 0.595
13382 measured reflections
2430 independent reflections
1631 reflections with *I* > 2σ(*I*)
*R*_int_ = 0.034

#### Refinement

*R*[*F* ^2^ > 2σ(*F* ^2^)] = 0.025
*wR*(*F* ^2^) = 0.069
*S* = 1.04
2430 reflections
125 parameters
H-atom parameters constrained
Δρ_max_ = 0.58 e Å^−3^
Δρ_min_ = −0.34 e Å^−3^

### 

Data collection: *APEX2* (Bruker, 2007 ▶); cell refinement: *SAINT* (Bruker, 2007 ▶); data reduction: *SAINT*; program(s) used to solve structure: *SHELXS97* (Sheldrick, 2008 ▶); program(s) used to refine structure: *SHELXL97* (Sheldrick, 2008 ▶); molecular graphics: *SHELXTL* (Sheldrick, 2008 ▶); software used to prepare material for publication: *SHELXTL*.

## Supplementary Material

Crystal structure: contains datablock(s) I, global. DOI: 10.1107/S1600536813000871/hy2613sup1.cif

Structure factors: contains datablock(s) I. DOI: 10.1107/S1600536813000871/hy2613Isup2.hkl

Additional supplementary materials:  crystallographic information; 3D view; checkCIF report

## Figures and Tables

**Table 1:** Selected bond lengths (Å)

Ru1—N1	2.086 (2)
Ru1—Br1	2.5439 (7)
Ru2—N2	2.083 (2)
Ru2—Br2	2.5378 (7)

**Fig. 1.**
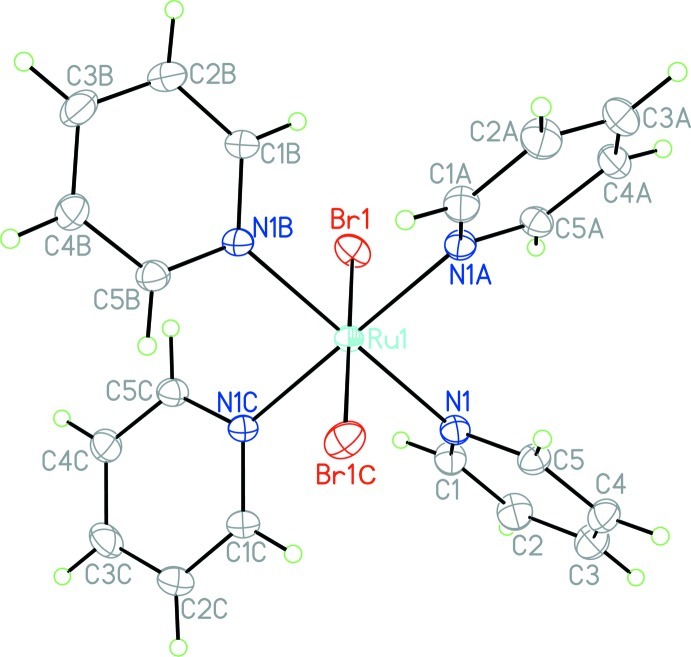
The molecular structure of the title compound, showing one of the two independent molecules. Displacement ellipsoids are drawn at the 50% probability level. [Symmetry codes: (A) x, 1/4-y, 1/4-z; (B) 1/4-x, y, 1/4-z; (C) 1/4-x, 1/4-y, z.]

**Fig. 2.**
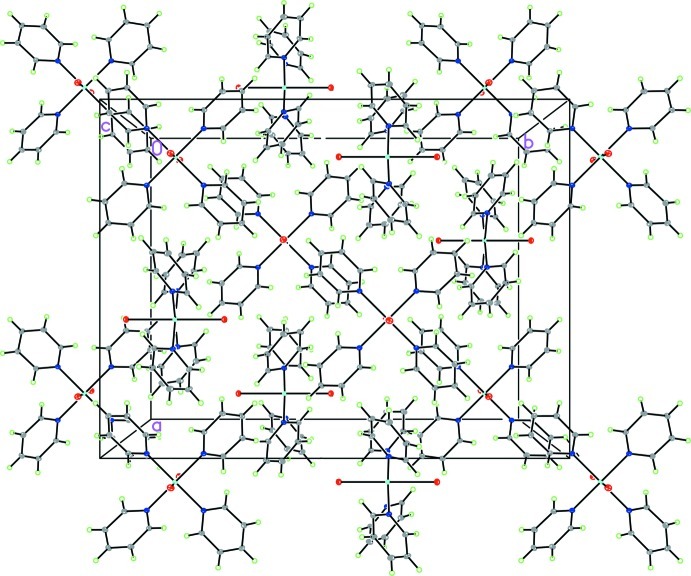
Packing diagram of the title compound in a unit cell, viewed along the c axis.

## supplementary crystallographic information

### Comment

Coordination chemistry of ruthenium complexes has been studied in last few decades because of their versatile and diverse applications in molecular catalysis (Pagliaro *et al.*, 2005) and bioinorganic chemistry (van Rijt & Sadler, 2009). As part of our long-standing interest in the ruthenium complexes with σ-donor ligands such as thiolate, pyridine and phosphine (Zhang *et al.*, 2005), we have investigated the reactivity of the starting ruthenium compounds such as RuCl_2_(PPh_3_)_3_, RuHCl(CO)(PPh_3_)_3_ and RuCl_2_(dmso)_4_ (dmso = dimethyl sulfoxide) with mono-, bi- and poly-dentate ligands (Wu *et al.*, 2009). Here we report the crystal structure of the mononuclear ruthenium(II) complex.

In the title complex, there are two independent complex molecules with a perpendicular arrangement. Each Ru^II^ atom is located on a 222 symmetry. No significant differences in bonding parameters between these two molecules are found. One of the molecular structures is depicted in Fig. 1. The coordination geometry of the Ru^II^ atom is octahedral with four pyridine N atoms and two Br atoms. The Ru—N bond lengths (Table 1) are in the range of those found in related structures of ruthenium(II) complexes retrieved from the Cambridge Structural Database (Allen, 2002). The Ru—Br bond lengths are comparable to those reported in other ruthenium(II)-bromide complexes such as [Ru_2_Br_2_(pz)(py)_8_][PF_6_]_2_.2DMF (pz = pyrazine, py = pyridine) [av. 2.5524 (4) Å] (Mirza *et al.*, 2003) and *trans*-[RuBr(py)_4_C(CN)_3_] [2.5453 (12) Å] (Zhang *et al.*, 2006). Two Br atoms are trans disposed as indicated by the Br—Ru—Br bond angle of 180°, as a result of symmetry requirements. Similar case was found in analogous complex *trans*-[RuCl_2_(py)_4_] (Wong & Lau, 1994).

### Experimental

To a THF solution (10 ml) of RuCl_2_(DMSO)_4_ (97 mg, 0.2 mmol) was added pyridine (63 mg, 0.8 mmol) and Br_2_ (32 mg, 0.2 mmol) under a nitrogen atmosphere. The reaction mixture was refluxed for 2 h, developing red. The solvent was evaporated in vacuo and the residue was washed with hexane. Recrystallization from CH_2_Cl_2_/hexane afforded red crystals of the title complex within two days (yield: 75 mg, 65 % based on Ru). Analysis, calculated for C_20_H_20_Br_2_N_4_Ru: C 41.61, H 3.49, N 9.70%; found: C 41.53, H 3.44, N 9.63%.

### Refinement

H atoms were placed in geometrically idealized positions and refined as riding atoms, with C—H = 0.93 Å and with *U*_iso_(H) = 1.2*U*_eq_(C).

### Crystal data

**Table:**

[RuBr_2_(C_5_H_5_N)_4_]	*F*(000) = 4512
*M**_r_* = 577.29	*D*_x_ = 1.781 Mg m^−^^3^
Orthorhombic, *F**d**d**d*	Mo *K*α radiation, λ = 0.71073 Å
Hall symbol: -F 2uv 2vw	Cell parameters from 2149 reflections
*a* = 16.830 (4) Å	θ = 2.2–26.4°
*b* = 22.032 (5) Å	µ = 4.45 mm^−^^1^
*c* = 23.221 (5) Å	*T* = 296 K
*V* = 8610 (3) Å^3^	Block, red
*Z* = 16	0.22 × 0.18 × 0.13 mm

### Data collection

**Table:**

Bruker APEXII CCD diffractometer	2430 independent reflections
Radiation source: fine-focus sealed tube	1631 reflections with *I* > 2σ(*I*)
Graphite monochromator	*R*_int_ = 0.034
φ and ω scans	θ_max_ = 27.5°, θ_min_ = 3.0°
Absorption correction: multi-scan (*SADABS*; Sheldrick, 1996)	*h* = −21→21
*T*_min_ = 0.441, *T*_max_ = 0.595	*k* = −28→28
13382 measured reflections	*l* = −29→26

### Refinement

**Table:**

Refinement on *F*^2^	Primary atom site location: structure-invariant direct methods
Least-squares matrix: full	Secondary atom site location: difference Fourier map
*R*[*F*^2^ > 2σ(*F*^2^)] = 0.025	Hydrogen site location: inferred from neighbouring sites
*wR*(*F*^2^) = 0.069	H-atom parameters constrained
*S* = 1.04	*w* = 1/[σ^2^(*F*_o_^2^) + (0.0292*P*)^2^ + 11.6805*P*] where *P* = (*F*_o_^2^ + 2*F*_c_^2^)/3
2430 reflections	(Δ/σ)_max_ < 0.001
125 parameters	Δρ_max_ = 0.58 e Å^−^^3^
0 restraints	Δρ_min_ = −0.34 e Å^−^^3^

### Special details

**Table:**

Geometry. All esds (except the esd in the dihedral angle between two l.s. planes) are estimated using the full covariance matrix. The cell esds are taken into account individually in the estimation of esds in distances, angles and torsion angles; correlations between esds in cell parameters are only used when they are defined by crystal symmetry. An approximate (isotropic) treatment of cell esds is used for estimating esds involving l.s. planes.
Refinement. Refinement of F^2^ against ALL reflections. The weighted R-factor wR and goodness of fit S are based on F^2^, conventional R-factors R are based on F, with F set to zero for negative F^2^. The threshold expression of F^2^ > 2sigma(F^2^) is used only for calculating R-factors(gt) etc. and is not relevant to the choice of reflections for refinement. R-factors based on F^2^ are statistically about twice as large as those based on F, and R- factors based on ALL data will be even larger.

### Fractional atomic coordinates and isotropic or equivalent isotropic displacement parameters (Å^2^)

**Table:**

	*x*	*y*	*z*	*U*_iso_*/*U*_eq_	
Ru1	0.1250	0.1250	0.1250	0.03562 (11)	
Ru2	0.6250	0.1250	0.1250	0.03634 (11)	
Br1	0.1250	0.009537 (19)	0.1250	0.05758 (14)	
Br2	0.6250	0.1250	0.015713 (18)	0.06143 (15)	
N1	0.03782 (12)	0.12460 (9)	0.18885 (9)	0.0409 (5)	
N2	0.71241 (13)	0.19195 (10)	0.12552 (9)	0.0428 (5)	
C1	−0.02375 (16)	0.08600 (14)	0.18763 (12)	0.0513 (7)	
H1	−0.0280	0.0594	0.1567	0.062*	
C2	−0.08052 (18)	0.08388 (16)	0.22953 (14)	0.0643 (9)	
H2	−0.1223	0.0564	0.2268	0.077*	
C3	−0.0757 (2)	0.12230 (16)	0.27562 (15)	0.0664 (9)	
H3	−0.1137	0.1216	0.3047	0.080*	
C4	−0.01284 (18)	0.16198 (15)	0.27768 (13)	0.0565 (8)	
H4	−0.0077	0.1887	0.3084	0.068*	
C5	0.04201 (16)	0.16198 (13)	0.23442 (11)	0.0463 (6)	
H5	0.0842	0.1891	0.2366	0.056*	
C6	0.77437 (16)	0.19016 (13)	0.16161 (13)	0.0513 (7)	
H6	0.7783	0.1577	0.1870	0.062*	
C7	0.83202 (18)	0.23371 (16)	0.16297 (15)	0.0655 (9)	
H7	0.8740	0.2304	0.1888	0.079*	
C8	0.8280 (2)	0.28178 (15)	0.12661 (16)	0.0721 (10)	
H8	0.8670	0.3117	0.1269	0.086*	
C9	0.76494 (19)	0.28493 (15)	0.08948 (16)	0.0656 (9)	
H9	0.7601	0.3175	0.0642	0.079*	
C10	0.70898 (16)	0.23981 (13)	0.08982 (13)	0.0509 (7)	
H10	0.6667	0.2424	0.0642	0.061*	

### Atomic displacement parameters (Å^2^)

**Table:**

	*U*^11^	*U*^22^	*U*^33^	*U*^12^	*U*^13^	*U*^23^
Ru1	0.0287 (2)	0.0399 (2)	0.0383 (2)	0.000	0.000	0.000
Ru2	0.0285 (2)	0.0431 (2)	0.0374 (2)	0.000	0.000	0.000
Br1	0.0600 (3)	0.0459 (2)	0.0668 (3)	0.000	−0.0158 (2)	0.000
Br2	0.0616 (3)	0.0806 (3)	0.0421 (2)	−0.0070 (2)	0.000	0.000
N1	0.0331 (11)	0.0465 (12)	0.0430 (12)	−0.0042 (10)	−0.0006 (9)	0.0007 (10)
N2	0.0326 (11)	0.0461 (12)	0.0497 (13)	0.0005 (9)	−0.0006 (10)	0.0022 (11)
C1	0.0398 (15)	0.0614 (18)	0.0526 (17)	−0.0119 (14)	−0.0014 (13)	0.0002 (14)
C2	0.0449 (18)	0.081 (2)	0.067 (2)	−0.0174 (16)	0.0047 (15)	0.0060 (18)
C3	0.0495 (18)	0.091 (2)	0.0590 (19)	−0.0018 (18)	0.0172 (15)	0.0106 (18)
C4	0.0482 (17)	0.072 (2)	0.0490 (17)	0.0020 (15)	0.0079 (13)	−0.0041 (15)
C5	0.0392 (15)	0.0486 (16)	0.0512 (17)	−0.0019 (12)	0.0030 (12)	−0.0028 (13)
C6	0.0382 (15)	0.0530 (17)	0.0627 (19)	0.0023 (13)	−0.0086 (14)	−0.0006 (14)
C7	0.0407 (17)	0.068 (2)	0.088 (2)	−0.0039 (15)	−0.0146 (17)	−0.0092 (18)
C8	0.0504 (19)	0.053 (2)	0.112 (3)	−0.0150 (15)	−0.001 (2)	−0.004 (2)
C9	0.0528 (19)	0.056 (2)	0.088 (2)	−0.0023 (15)	0.0069 (18)	0.0133 (18)
C10	0.0380 (15)	0.0549 (18)	0.0597 (18)	−0.0009 (13)	0.0018 (13)	0.0077 (14)

### Geometric parameters (Å, º)

**Table:**

Ru1—N1^i^	2.086 (2)	C1—H1	0.9300
Ru1—N1^ii^	2.086 (2)	C2—C3	1.367 (5)
Ru1—N1	2.086 (2)	C2—H2	0.9300
Ru1—N1^iii^	2.086 (2)	C3—C4	1.373 (4)
Ru1—Br1	2.5439 (7)	C3—H3	0.9300
Ru1—Br1^ii^	2.5439 (7)	C4—C5	1.364 (4)
Ru2—N2^iv^	2.083 (2)	C4—H4	0.9300
Ru2—N2	2.083 (2)	C5—H5	0.9300
Ru2—N2^i^	2.083 (2)	C6—C7	1.365 (4)
Ru2—N2^v^	2.083 (2)	C6—H6	0.9300
Ru2—Br2	2.5378 (7)	C7—C8	1.356 (5)
Ru2—Br2^v^	2.5378 (7)	C7—H7	0.9300
N1—C1	1.341 (3)	C8—C9	1.370 (5)
N1—C5	1.343 (3)	C8—H8	0.9300
N2—C6	1.339 (3)	C9—C10	1.369 (4)
N2—C10	1.342 (3)	C9—H9	0.9300
C1—C2	1.365 (4)	C10—H10	0.9300
			
N1^i^—Ru1—N1^ii^	179.52 (12)	C6—N2—C10	116.3 (2)
N1^i^—Ru1—N1	90.60 (12)	C6—N2—Ru2	122.20 (18)
N1^ii^—Ru1—N1	89.40 (12)	C10—N2—Ru2	121.48 (18)
N1^i^—Ru1—N1^iii^	89.40 (12)	N1—C1—C2	123.2 (3)
N1^ii^—Ru1—N1^iii^	90.60 (12)	N1—C1—H1	118.4
N1—Ru1—N1^iii^	179.52 (12)	C2—C1—H1	118.4
N1^i^—Ru1—Br1	90.24 (6)	C1—C2—C3	119.7 (3)
N1^ii^—Ru1—Br1	90.24 (6)	C1—C2—H2	120.2
N1—Ru1—Br1	89.76 (6)	C3—C2—H2	120.2
N1^iii^—Ru1—Br1	89.76 (6)	C2—C3—C4	117.9 (3)
N1^i^—Ru1—Br1^ii^	89.76 (6)	C2—C3—H3	121.1
N1^ii^—Ru1—Br1^ii^	89.76 (6)	C4—C3—H3	121.1
N1—Ru1—Br1^ii^	90.24 (6)	C5—C4—C3	119.7 (3)
N1^iii^—Ru1—Br1^ii^	90.24 (6)	C5—C4—H4	120.2
Br1—Ru1—Br1^ii^	180.0	C3—C4—H4	120.2
N2^iv^—Ru2—N2	179.34 (11)	N1—C5—C4	123.0 (3)
N2^iv^—Ru2—N2^i^	89.84 (12)	N1—C5—H5	118.5
N2—Ru2—N2^i^	90.16 (12)	C4—C5—H5	118.5
N2^iv^—Ru2—N2^v^	90.16 (12)	N2—C6—C7	123.2 (3)
N2—Ru2—N2^v^	89.84 (12)	N2—C6—H6	118.4
N2^i^—Ru2—N2^v^	179.34 (11)	C7—C6—H6	118.4
N2^iv^—Ru2—Br2	90.33 (6)	C8—C7—C6	120.0 (3)
N2—Ru2—Br2	90.33 (6)	C8—C7—H7	120.0
N2^i^—Ru2—Br2	89.67 (6)	C6—C7—H7	120.0
N2^v^—Ru2—Br2	89.67 (6)	C7—C8—C9	118.0 (3)
N2^iv^—Ru2—Br2^v^	89.67 (6)	C7—C8—H8	121.0
N2—Ru2—Br2^v^	89.67 (6)	C9—C8—H8	121.0
N2^i^—Ru2—Br2^v^	90.33 (6)	C10—C9—C8	119.5 (3)
N2^v^—Ru2—Br2^v^	90.33 (6)	C10—C9—H9	120.2
Br2—Ru2—Br2^v^	180.0	C8—C9—H9	120.2
C1—N1—C5	116.5 (2)	N2—C10—C9	123.0 (3)
C1—N1—Ru1	122.09 (18)	N2—C10—H10	118.5
C5—N1—Ru1	121.38 (17)	C9—C10—H10	118.5

Symmetry codes: (i) *x*, −*y*+1/4, −*z*+1/4; (ii) −*x*+1/4, −*y*+1/4, *z*; (iii) −*x*+1/4, *y*, −*z*+1/4; (iv) −*x*+5/4, −*y*+1/4, *z*; (v) −*x*+5/4, *y*, −*z*+1/4.
